# Implementation and optimization of hydraulic wave energy generation system

**DOI:** 10.1371/journal.pone.0293209

**Published:** 2024-02-15

**Authors:** Zhigang Liu, Shi Liu, Wen Chen, Yi Yang, Guoliang Feng

**Affiliations:** 1 China Southern Power Grid Technology Co., Ltd., Guangzhou, Guangdong, China; 2 College of Automation Engineering, Northeast Electric Power University, Jilin, Jilin, China; NUST: National University of Sciences and Technology, PAKISTAN

## Abstract

Wave energy is one of the primary sources of marine energy, representing a readily available and inexhaustible form of renewable clean energy. In recent years, wave energy generation has garnered increasing attention from researchers. To study wave energy generation technology, we have constructed a real wave energy generation system and designed wave simulation and hydraulic energy storage systems. The wave simulation system is mainly composed of a frequency converter and an electric boost pump, while the hydraulic energy storage system consists of a hydraulic control unit and hydraulic motors. Corresponding mathematical models have been established to investigate the characteristics of wave energy generation. Specifically, a mathematical model for wave input using the double-parameter JONSWAP wave spectrum has been created for wave simulation in the wave simulation system. For the hydraulic energy storage system, known as the Power Take Off (PTO) system, mathematical models have been developed for double-acting hydraulic cylinders, energy storage devices, and precise displacement hydraulic motors, taking into consideration fluid Reynolds numbers and leakage. During the generation of wave energy, there is a problem of prolonged power interruption when wave conditions are unfavorable, which hinders continuous power generation. To address this issue, a system structure with an energy storage unit and two parallel generator sets, as well as a power operation optimization scheme, have been proposed. This system structure and optimization approach efficiently and reasonably utilize wave energy, achieving the goal of uninterrupted power supply in the hydraulic wave energy generation system.

## 1 Introduction

As energy demand continues to rise, there is a steady increase in the share of sustainable and renewable energy sources. The oceans house various forms of renewable energy, including tidal, temperature, salt, and wave energy. Experts have increasingly emphasized and researched wave energy in recent years due to its significant development potential. Research indicates that approximately 1200 TWh of tidal energy, 44,000 TWh of temperature difference energy, 1650 TWh of salt difference energy, and 29,500 TWh of wave energy are potentially exploitable. Projections suggest that ocean energy capacity could reach 352 GW by 2050 [1]. Wave energy exhibits greater spatial concentration and predictability compared to wind and solar energy. For instance, a 1 km2 expanse of sea surface has the potential to generate approximately 200 MW of wave energy [2], suggesting that the global wave energy reserve could be as extensive as 2.5 TW.

The study of wave power generation dates back to the 1970s. The wave energy power generation device functions by converting the oscillating and rocking motion of the floating body under wave action, the change of wave pressure, or the wave climbing along the coast into the kinetic and potential energy of water. Currently, countries such as China, Japan, Britain, Ireland, Norway, Spain, Portugal, Sweden, Denmark, Australia, and the United States have established wave power generation devices floating on the sea or fixed on the coastline [3, 4]. In recent years, wave power generation devices have experienced rapid development and are rapidly progressing towards commercialization and practical application.

With the advancement of wave energy technology, a variety of systems for converting wave energy to electrical energy have been developed [5–7]. The machinery used in the process of converting wave energy into electricity is collectively known as wave energy converters, which can be categorized into Oscillation Water Column(OWC), Overtopping Devices, and Wave Activated Bodies according to the principle of operation. The OWC utilizes the motion of waves to drive the moving part of the device in reciprocating motion, which drives the mechanical system or hydraulic system, which further drives the generator to generate electricity [8]. Overtopping technology utilizes a water circuit to introduce waves into a high level reservoir, where the outflow of water from the reservoir’s downward-extending outlet pipe generates gravitational potential energy, which drives a turbine generator to produce electricity [9]. Oscillation buoy type wave energy generators are one of the more commonly used generators [10]. However, due to the reciprocity and instability of the airflow within the pneumatic device, the conversion efficiency of the wave energy converter cannot be significantly enhanced [11]. Different types of wave energy generators are optimized in various ways, with the goal of increasing the device’s overall generating capacity. From gathering wave energy to generating electrical energy, wave energy power generation devices are influenced by a number of elements, including offshore wave conditions, component type, device construction, and so on.

Wave energy’s changing nature causes substantial changes in output power and intermittent generation [12, 13]. There are a number of different technologies and devices included in a wave energy generation system, each with its own specific parameters. The design parameters of various types of wave energy converters have a considerable impact on power generation in wave energy converters. Lawrence V et al. [14] investigated the wave energy converter location problem and discovered that the best distance between two devices reduces as uncertainty increases. Liguo Wang and Ringwood [15] researched optimal raft module dimensions and discovered the best design solution for a certain sea state situation. Sarkar et al. [16] discuss the hydrodynamics of the modular concept of wave energy device-the Oscillating Wave Surge Converter. Using a tightly spaced modular system to generate several resonances has the ability to leverage these resonances to capture more energy, improving the overall power output. However, the ability to achieve an optimized wave energy generation system by optimizing the structure is limited, so control techniques are again introduced to optimize the system. Guo et al. [17] and Zheng et al. [18] proposed nonlinear absorption devices with nonlinear stiffness and damping mechanism respectively. In an oscillating water column wave energy power plant, Ebrahim Zarei et al. [19] proposed a model predictive current control for a three-phase four-switch converter coupled to a surface permanent magnet synchronous generator. When compared to existing control methods for this type of power plant, the suggested control method minimizes the current reference tracking error and is suited for power decoupling systems used in wave energy converters.

Moretti Giacomo et al. [20] constructed and presented a dielectric elastomer generator-based wave energy converter with a peak power output of up to 3.8 W shown experimentally via a wave flume, corresponding to a maximum power of several hundred kilowatts under real sea conditions. The acquired results demonstrated the tangible possibility of developing a DEG-based WEC device for large-scale electrical energy production. Yapo Wang et al. [21] proposed a combined concept consisting of a 5MW braceless semisubmersible floating offshore wind turbine (FOWT) and an annular-type wave energy converter. The kinematic mechanism of the structural response is realized by altering the interface stiffness and damping coefficients between the WEC and the semi-submersible float. Noad et al. [22] investigated the performance and optimal configuration of WEC arrays. In addition, a numerical optimization method was used to determine the optimal parameters of individual WEC devices and it was concluded that the gap length is the key factor affecting the power output. Yongxing Zhang et al. [23] used the analytic hierarchy process (AHP) to develop a comprehensive multi-index model to evaluate the existing water resource utilization efficiency from five aspects: energy acquisition, technical cost economy, reliability, environmental friendliness, and adaptability. The results demonstrated that the MDWEC (multi-directional wave energy converter) has good comprehensive performance and a wide application range in the field of wave energy utilization and development.

Ting Li et al. [24] suggested a hybrid AC-DC transmission system based on modular multilevel converter (modular multilevel converter) to formulate a composite control approach to govern the power quality of wave energy generation. The method is shown to efficiently limit the three-phase voltage drop of the power supply network while assuring high grid accuracy by evaluating voltage harmonic distortion and current distortion. However, only the power quality issue of transient voltage dips was evaluated, and no other difficulties were investigated. J. F. Pan et al. [25] proposed a complementary compensation power generation scheme using two asymmetric bilateral linear switched reluctance generators (ABLSRGs) to address the issues of low output energy and poor robustness in wave power generation systems based on single linear generator schemes. Experiments showed that the proposed complementary power generation control scheme could ensure both voltage control accuracy and system robustness.

The Power Take-Off systems (PTOs) in wave energy generation systems can be categorized into two main types: electric and hydraulic [26]. Electric PTOs convert wave energy directly into electrical energy, mainly using linear generators and rotary generators with gearboxes [27]. Hydraulic PTOs convert wave energy into hydraulic energy, which drives a turbine to generate electricity [28]. Hydraulic PTOs are more adaptable to powerful waves at low speeds than electric PTOs, and they are smaller and lighter, less expensive, and easier to install and maintain [29]. Dong Wang and Lu [26] designed a hydraulic energy storage and conversion system and detailed modeled it to evaluate the system’s control strategy and efficiency. The connection between system pressure, storage capacity, and cost was investigated. Hong Gao et al. [30] studied the impacts of hydraulic power output parameters and generator damping on pumping energy, output power, and efficiency in a wave energy hydraulic conversion system with vibrating harvester. The hydraulic parameters are optimized based on genetic algorithm to maximize the motor output power for three working conditions: conical, cylindrical and hemispherical for a given sea state. The effect of parameters such as working area of the hydraulic cylinder piston, motor displacement, and hydraulic conversion efficiency on the output of the motor was investigated. McDonald S. P. et al. [31] compared two power converter topologies for power PTOs. This EPTO replaces the conventional MPTO and generator with a permanent magnet linear generator directly coupled to the WEC. Comparative topologies include current-source converters or voltage-source converters as generator interfaces in combination with DC-DC converters and energy storage systems. The results show that the current source converter topology is advantageous at higher switching frequencies. Wei Zhang [32] examined the dynamic response of a wave energy device’s hydraulic transmission system under random settings, developed a mathematical model of hydraulic transmission, and clarified the factors linked to electrical energy output. The experimental results show that changing the load, accumulator charging pressure, and throttle opening can significantly increase the efficiency and stability of the output electrical energy. Because of the significant volatility of wave energy, YH Yu et al. [33] believed that wave energy converter components should be able to bear loads many times greater than the average load. The hydraulic PTO model is studied in detail and combined with a time domain fluid dynamics model (WEC-Sim). The problem of the unit’s fluctuating maximum power was greatly decreased by employing three different power smoothing methods, namely, energy storage, pressure release mechanism, and power-based set point control method, all of which trade off power output and fluctuation.

With the development of artificial intelligence technology, in recent years, for the power optimization of wave energy converter, some optimization methods based on artificial intelligence technology have been proposed [34]. As the complexity hydrodynamic interactions among WECs, the evaluation of each parameter setting is computationally expensive. To tackle the challenge of optimising the positions of WECs in a wave farm, Neshat et al. [35] propose a novel multi-swarm cooperative co-evolution algorithm and exhibit better performance compared with other meta-heuristics methods. He et al. [36] present a parameter optimization method for an oscillating buoy-type wave energy converter from the perspective of submerged buoy volume. the differential evolution algorithm and linear potential flow theory are used to investigate the effects of submerged buoy volume on the optimal power capture.

Both early and modern wave power generation technologies generate power from the energy of the waves, and the randomness of the waves affects the quality of the generated power. Ensuring the stability and reliability of wave power generation and improving its power quality are key and difficult aspects of wave power generation technology.

In order to study the wave power generation, we established the simulation model of each module of the wave power generation system, and designed the wave power generation experimental system. The mathematical models of wave input, double acting hydraulic cylinder, accumulator and hydraulic motor are established. During the research, it is found that the performance of the energy storage wave energy generator system is greatly affected by the wave conditions and system parameters, which is prone to the problem of power generation interruption. In order to solve this problem, we further designed the topology of a wave energy power generation system with one accumulator and two generators, and studied how to solve the problem of unreasonable energy utilization by controlling the energy distribution between the two units, so that the accumulator pressure changes more smoothly and the power generation rate is uninterrupted.

The paper is organized as follows. Section 2 introduces the establishment of hydraulic wave energy power generation model. In Section 3, one accumulator and two unit system is proposed to mitigate power interruption. In Section 4, the experiments are exploited to demonstrate the advantages of the proposed model. Section 5 provides some helpful conclusions.

## 2 Modeling of hydraulic wave energy power generation system

**Fig 1:**
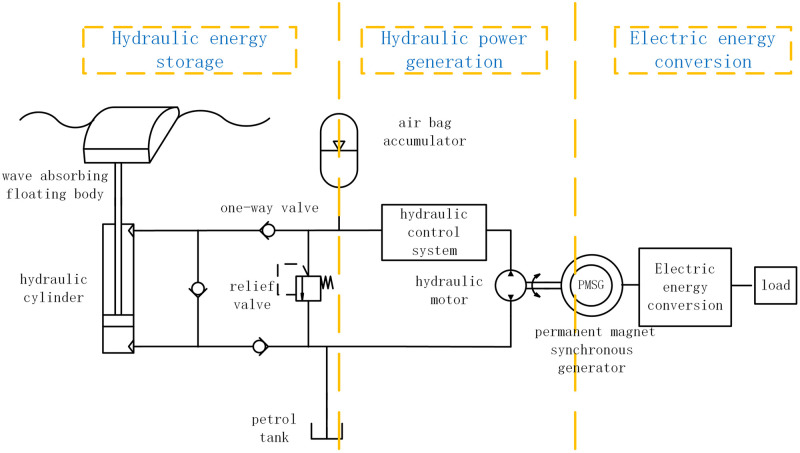
The structure of hydraulic wave energy power generation system.

The wave energy power generation system operates on the principle of wave energy conversion into hydraulic energy. This is accomplished through the use of a wave-absorbing floating body and hydraulic cylinder that stores the hydraulic energy in an accumulator. The hydraulic cylinder continues to operate, resulting in the incremental rise of the accumulator pressure. Once the pressure surpasses the upper threshold, the hydraulic control system initiates the hydraulic motor, causing the generator to produce electrical power in conjunction with the motor. If the wave conditions are favorable, the generator will continue to produce power continuously. Conversely, if the wave conditions are suboptimal, the accumulator pressure will decrease, and once it falls below the lower threshold, the hydraulic control system will disengage the hydraulic motor, transitioning the system into the next stage of energy storage and power generation. A schematic of the system structure is presented in Fig 1.

### 2.1 Wave model

In all sea states, the JONSWAP spectrum are employed to simulate waves [37].

$$\begin{array}{l} S(w)=\frac{5}{16}\cdot H_{S}^{2}\cdot w_{p}^{4}\cdot w^{-5}\cdot e^{-1.25}{\left(\frac{w}{w_{p}}\right)}^{-4}\cdot \left(1-0.287\cdot \text{ln}\;\gamma \right)\cdot \gamma^{e-0.5}\cdot {\left(\frac{w-w_{p}}{{\sigma w}_{p}}\right)}^{2}\\ w_{p}=\frac{2\pi }{T_{P}} \end{array}$$ (1)

Where, *H*_*s*_ is significant wave height and its unit is *m*, *w*_*p*_ stands spectral peak frequency and its unit is *rad*/*s*, w represents wave frequency and its unit is *rad*/*s*, γ is spectral peak factor, *σ* is peak shape parameter. If *w* ≤ *w*_*p*_, *σ* = 0.07, while *w* > *w*_*p*_, *σ* = 0.09. *T*_*P*_ stands peak period and its unit is *s*.

Assuming the wave propagation is unidirectional, the problem is simplified to a two-dimensional linear wave. According to the linear wave theory, the two-dimensional linear waves are comprised of an infinite number of linear regular waves, characterized by different amplitudes, periods, and wavelengths. Inherently, the phases of each regular wave are random. Consequently, the expression for the height of an irregular wave can be derived.


$$H\left(t\right)=\Sigma_{i=1}^{\infty }a_{i}\cdot \text{cos}\left(k_{i}\cdot x-w_{i}\cdot t+\varphi_{i}\right)$$ (2)

Considering that the frequency cannot be infinite, we have

$$H\left(t\right)\approx \sum_{i=1}^{N}a_{i}\cdot \text{cos}\left(k_{i}\cdot x-w_{i}\cdot t+\varphi_{i}\right)$$ (3)

Since only the heave motion of the system is considered and the displacement of the still water surface is not considered, set x = 0. The amplitude of the regular wave of each constituent unit can be taken as

$$a_{i}=\sqrt{2\cdot S\left(w_{i}\right)\cdot \Delta w_{i}}$$ (4)

where, *a*_*i*_ is the amplitude of the ith regular wave. w_i_ is the frequency of the ith regular wave. S(w) is the JONSWAP wave energy spectrum formula. Δw_i_ is the difference between adjacent frequencies.

As it is difficult to integrate within the frequency range of [0, ∞], it is generally believed that the energy of JONSWAP wave energy spectrum is concentrated near the spectral peak frequency, so the calculated frequency range is [*w*_*L*_, *w*_*H*_]. In order to obtain the random wave waveform of regular wave superposition, this paper uses the equal frequency method to divide the calculated frequency range into *k* equal parts, and the frequency interval is:

$$\Delta w=\frac{w_{H}-w_{L}}{K}$$ (5)

For each frequency interval, w_i_, any frequency value can be selected from within the range of that interval. If *i* < *K*, then $\frac{w_{i}-w_{L}}{\Delta w}\in [i-1,i)$. if *i* = *K*, then *w*_*K*_ ∈ [*K* − 1, *K*]. The phase angle *ϕ*_i_ of the wave elevation can take any value in [0,2π].

### 2.2 Double acting hydraulic cylinder

**Fig 2:**
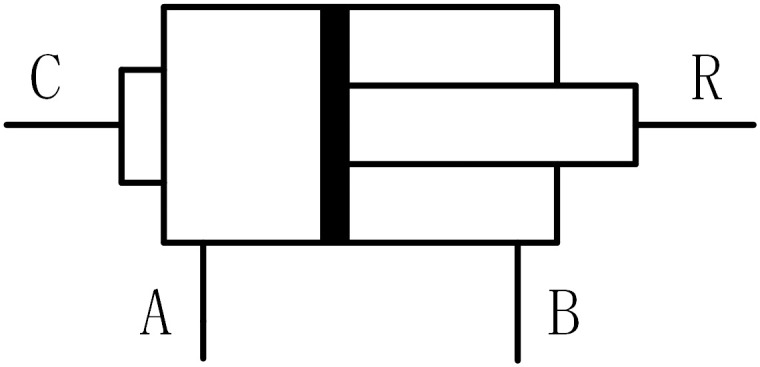
Hydraulic cylinder structure.

In view of the necessity to replicate the fundamental operation of hydraulic cylinders with improved numerical efficiency, the current study postulates the neglect of pertinent factors, namely fluid compressibility, friction, and leakage. To entirely suppress any potential oscillation at the end of the stroke, a completely inelastic hard stop is employed. The hydraulic cylinder configuration is depicted in Fig 2.

The model can be described by the following equation [38, 39]:

$$\begin{array}{c} F=A_{A}P_{A}-A_{B}P_{B}-F_{C}\\ Q_{A}=A_{A}\upsilon \\ Q_{B}=A_{B}\upsilon \\ \upsilon =\upsilon_{R}-\upsilon_{C} \end{array}$$ (6)

$$\begin{array}{c} F_{C}=\left\{\begin{array}{l} \left(x-x_{E}\right)K_{P}\upsilon ,if\;x>x_{E},\upsilon >0\\ \left(x-x_{E}\right)K_{P}\upsilon ,if\;x<x_{E},\upsilon <0\\ 0 \end{array}\right.\\ x_{E}=S-x_{0}\\ x_{R}=-x_{0} \end{array}$$ (7)

Where, *F* is cylinder rod thrust; *υ* is the cylinder rod speed; *υ*_*R*_, *υ*_*C*_ Absolute speed of cylinder rod and cylinder body respectively; *A*_*A*_ is the area of side a piston; *A*_*B*_ is the piston area at side B; *P*_*A*_ is the pressure at cylinder port a; *P*_*B*_ is the pressure at cylinder port B; *Q*_*A*_ is the flow into the cylinder through port a; *Q*_*B*_ is the flow from the cylinder through port B; *x* is the piston position; *x*_0_ is the initial distance between the piston and cover a; *F*_*C*_ is the hard stopping force; *x*_*E*_ is the absolute distance that the piston extends from the initial position; *x*_*R*_ is the absolute distance that the piston retracts from the initial position; *K*_*P*_ is the penetration coefficient; *S* is the piston stroke.

The motion characteristics of the double acting hydraulic cylinder match the wave motion characteristics, so the double acting hydraulic cylinder model is adopted.

### 2.3 Air bag accumulator

**Fig 3:**
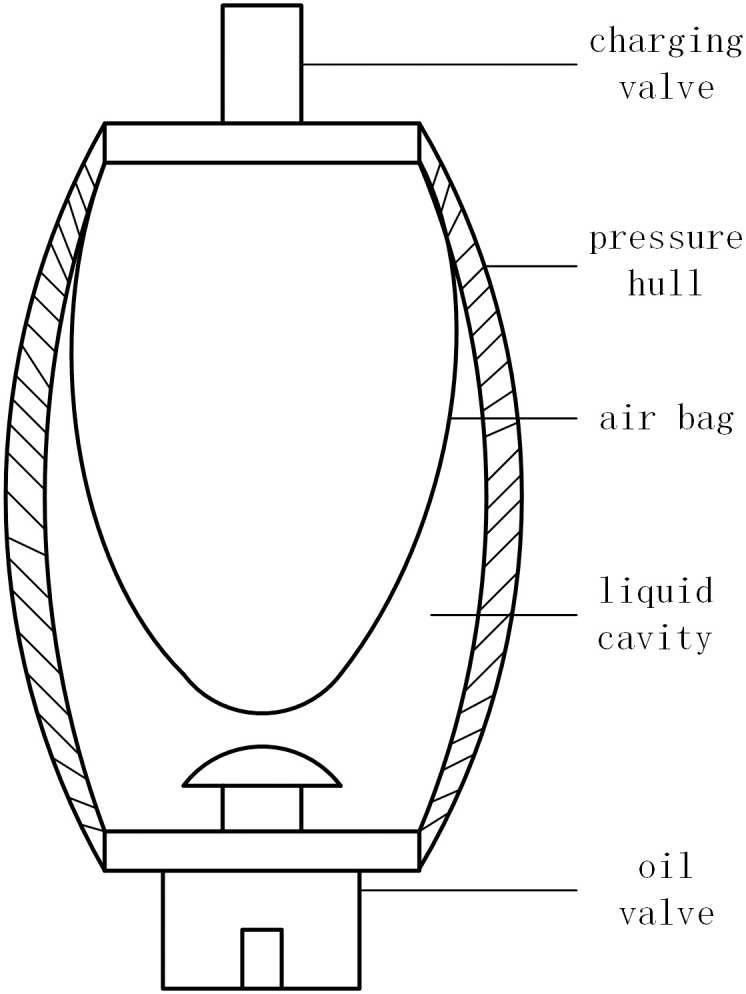
Structure of airbag accumulator.

The air bag accumulator is well-suited for the high-pressure conditions encountered in wave energy generation. The structure of the air bag accumulator can be depicted in Fig 3.

The hydraulic air bag accumulator meets Boyle’s Law [40]:

$$P_{\text{c0}}V_{\text{c}0}^{\gamma }=P_{c}V_{c}^{\gamma }$$ (8)

Where, *γ* is the adiabatic index, a dimensionless quantity. It is typically characterized by a value of 1.4 and associated with a value of 1 in the isothermal process. *P*_*c*0_ is the initial pressure of the air chamber; *V*_*c*0_ is the initial volume of the air chamber; *P*_*c*_ is the chamber pressure; *V*_*c*_ is the volume of the air chamber.

The accumulator operates on the principle that fluid enters the accumulator and compresses the gas to store energy when the flow pressure at the inlet exceeds the gas pressure in the airbag. The power generation process involves two processes: oil filling (gas compression) and oil discharge (gas expansion). The gas state in the accumulator’s gas chamber undergoes adiabatic change within 3 seconds, while the gas state change process after 3 seconds is isothermal.

According to Boyle’s law, the formula for describing the air bag accumulator can be defined in the following form,

$$\begin{array}{c} \left(P_{pr}+P_{air}\right)V_{acc}=\left(P_{acc}+P_{air}\right)\left(V_{acc}-V_{f}\right)\\ P_{acc}=\frac{\left(P_{pr}+P_{air}\right)V_{acc}}{V_{acc}-V_{f}}-P_{air} \end{array}$$ (9)

Where, *V*_*acc*_ is accumulator volume (m3); *P*_*pr*_ is pre inflation pressure (Pa); *P*_*air*_ is atmospheric pressure (Pa); *V*_*f*_ is the volume of hydraulic oil in the fluid chamber (m3); *P*_*acc*_ is the accumulator pressure (Pa), and the pressure of the gas cavity is the same as that of the liquid cavity. Under the same accumulator, *V*_*acc*_ *P*_*pr*_, *P*_*air*_ are fixed values, and the accumulator pressure *P*_*acc*_ is a function of the volume of hydraulic oil *V*_*f*_ in the fluid chamber.

For a given accumulator, *V*_*acc*_, *P*_*pr*_, *P*_*air*_ are constant values, so the accumulator pressure *P*_*acc*_ is a function of the volume of hydraulic oil *V*_*f*_ in the fluid chamber. By controlling the volume of hydraulic oil in the accumulator, it is possible to regulate the pressure of the accumulator.

The volume of hydraulic oil in the accumulator is influenced by two factors. Firstly, the hydraulic oil flow into the hydraulic cylinder, and secondly, the hydraulic oil flow from the hydraulic motor to the oil tank. It should be noted that a portion of the hydraulic oil flows into the oil tank from the overflow valve when the pressure is excessively high. If the two flows are maintained in equilibrium and the volume of hydraulic oil in the accumulator is constant, the accumulator pressure will remain stable.

### 2.4 Hydraulic motor

The hydraulic motor is an essential component in hydraulic power generation systems, responsible for converting energy. In order to meet the requirements of high working speed and reliability, the axial piston quantitative motor is commonly used. This motor offers numerous advantages, including stable and reliable performance, high speed capability, low moment of inertia, easy starting and braking, and high energy conversion efficiency. The flow equation for the hydraulic motor can be expressed as follows [39, 41].

$$\begin{array}{l} Q_{m}=Q_{m0}+Q_{ml}\\ \left\{\begin{array}{l} Q_{m0}=\frac{q_{m}n_{m}}{60}\\ Q_{ml}=C_{ml}\Delta p \end{array}\right. \end{array}$$ (10)

Where: *Q*_*m*0_ is the theoretical flow of hydraulic motor and its unit is m^3^/s; *Q*_*ml*_ is the leakage flow of hydraulic motor and its unit is m^3^/s. *q*_*m*_ is the displacement of hydraulic motor and its unit is m^3^/r. *n*_*m*_ is the rotational speed of the hydraulic motor and its unit is r/min. *C*_*ml*_ is the leakage coefficient of hydraulic motor. Δ*p* is the pressure difference between the inlet and outlet of the hydraulic motor, which is controlled by the hydraulic autonomous system.

Then the flow equation of the hydraulic motor *Q*_*m*_ is as follows.


$$Q_{m}=\frac{q_{m}n_{m}}{60}+C_{ml}\Delta p$$ (11)

In reality, the flow rate *Q*_*m*_ through the hydraulic motor is equivalent to the hydraulic flow rate *Q*_*acc*_ during the energy release process of the accumulator. As such, when the leakage coefficient is disregarded, the two flows can be considered equal.


$$Q_{acc}=Q_{m}=\frac{q_{m}n_{m}}{60}$$ (12)

Given that atmospheric pressure *p*_*air*_ is significantly lower than the working pressure, and the temperature of the accumulator remains constant, the atmospheric pressure can be disregarded. Utilizing the ideal gas state equation, the gas state equation under real-time pressure of the accumulator can be determined through the simultaneous utilization of the accumulator flow and volume relationship formula.

$$p(t)(V_{acc}-V_{f0}+\int_{0}^{t}\frac{q_{m}\cdot n_{m}}{60}dt)=V_{acc}p_{pr}$$ (13)

Where: *V*_*f*0_ is the volume of hydraulic oil in the initial liquid chamber (m^3^). The agreed hydraulic flow is positive when it flows out of the accumulator.

Thus, the real-time pressure equation of the accumulator can be obtained.


$$p(t)=\frac{V_{acc}(p_{pr}+p_{air})}{\left(V_{acc}-V_{f0}+\int_{0}^{t}\frac{q_{m}\cdot n_{m}}{60}dt\right)}-p_{air}$$ (14)

According to the power calculation and power balance formula of the hydraulic motor, the input power *P*_*in*_ and output power *P*_*out*_ of the hydraulic motor are

$$\left\{\begin{array}{l} P_{in}=Q_{m}\cdot \Delta p=\frac{q_{m}n_{m}}{60}\Delta p\\ P_{out}=\eta_{M}\eta_{V}P_{in}=T_{m}\omega_{m} \end{array}\right.$$ (15)

Where *η*_*M*_ and *η*_*V*_ are the mechanical efficiency and volumetric efficiency of the hydraulic motor respectively; *T*_*m*_ is the output torque of the hydraulic motor (N·m).

The relationship between rotational speed and mechanical angular speed is as follows

$$n_{m}=\frac{60}{2\pi }\omega_{m}$$ (16)

If the leakage coefficient is ignored, the output mechanical torque of the hydraulic motor can be further expressed as

$$T_{m}=\frac{q_{m}\eta_{M}\eta_{V}\Delta p}{2\pi }=k\Delta p$$ (17)

Where, *k* = *q*_*m*_*η*_*M*_*η*_*V*_/2*π* is the parameter coefficient of the hydraulic motor system. The above formula shows that the generator input torque (the hydraulic motor output mechanical torque) *T*_*m*_ is determined by the hydraulic motor efficiency, the pressure difference between the inlet and outlet of the hydraulic motor, and the displacement of the hydraulic motor. Simultaneously, owing to the fact that the hydraulic motor’s efficiency and displacement are both constant values, the resultant output torque of the hydraulic motor is directly proportional to the pressure of the accumulator. This correlation results in the formation of a linear functional relationship between the two variables. That is

$$T_{m}=k\Delta p$$ (18)

## 3 One accumulator and two unit system

**Fig 4:**
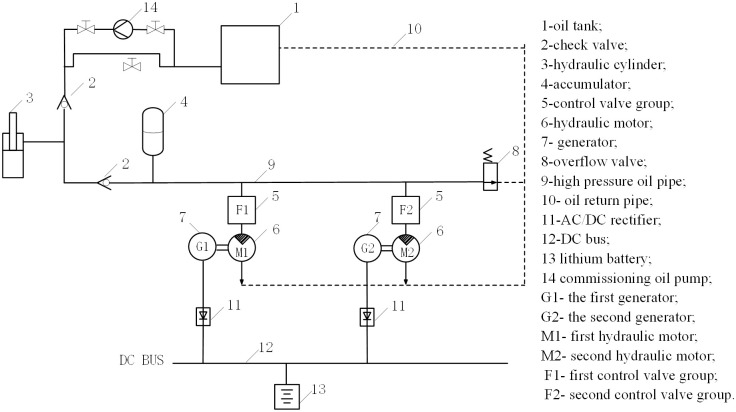
The topology structure of the power generation system.

The traditional wave energy generation model only has a single unit. This structure can lead to long periods of power interruption when the wave conditions are not good, and cannot provide continuous uninterrupted power supply. To address this issue, we propose a strategy of parallel operation of an energy storage device and two generator sets to achieve continuous uninterrupted power supply for a sustainable wave energy generation system. The new wave energy generation system includes a wave absorbing float, a hydraulic system, an accumulator, a control valve block, a hydraulic motor generator set, and a hydraulic oil tank. The topology structure of the power generation system is shown in Fig 4.

In hydraulic autonomous conditions without any operational control, the parallel operation scheme is still susceptible to power interruption. The primary cause of power interruption is inefficient energy utilization. The current study aims to address this issue by regulating the energy distribution of the two units. Based on this problem, this paper proposes a power operation optimization approach to mitigate power interruption and enhance energy utilization. In light of an assessment of the challenges encountered in the autonomous operational mode, a comprehensive investigation has been conducted to explore a coordinated operation approach that enhances the seamless and uninterrupted power output of wave energy power generation systems.

In the commissioning stage, the hydraulic system is pressurized by the commissioning oil pump, and the function of the amount of electricity *W*(*P*) that can be released by the accumulator under any initial pressure (*P*_min_ ~*P*_max_) is tested, and the amount of electricity *W*_*x*_ that can be generated by the accumulator pressure *P*_max_ is obtained.


$$W(P)=\int_{P\text{min}}^{P}Q(t)dt$$ (19)

Generator G1 generates electricity with a power of *Q*_1_, while generator G2 generates electricity with a power of *Q*_2_. Using the smallest generator G1 configured by the system, the maximum time *T*_*x*_ that the accumulator can maintain the auxiliary power is measured, which should be close to the following values.


$$T_{x}\approx \frac{W_{x}}{Q_{1}}$$ (20)

After the wave energy power generation system is put into operation, it starts to operate in autonomous mode. Calculate the average power *Q*_*ave*_ in the latest period 3*T*_*x*_ in real time.

$$Q_{ave}=\frac{1}{3T_{x}}\left[{\text{(W(P}}_{\text{3Tx}}{\text{)-W(P}}_{\text{0}}\text{)}+\int_{0}^{3T_{x}}Q(t)dt\right]$$ (21)

Where, W(P_3Tx_) indicates the electric quantity that can be generated by the accumulator at the time 3*T*_*x*_ of real-time calculation, W(P_0_) is the electric energy generated by the accumulator at time 0 is calculated in real time, *Q*(*t*) represents the total power generated.

Dynamic pressure setting and time parameters used in real-time calculation of electronic control working mode.

$$P_{\text{min}}^{*}=P_{\text{min}},P_{\text{max}}^{*}=P_{\text{max}}$$ (22)

$$P_{1}^{*}=P_{\text{min}}^{*}+\text{min}\left(\text{max}\left(L_{p1},k_{1}*Q_{1}/Q_{ave}\right),H_{p1}\right)$$ (23)

$$P_{2}^{*}=P_{\text{max}}^{*}-\text{min}\left(\text{max}\left(L_{p2},k_{2}*Q_{ave}/\left(Q_{1}+Q_{2}\right)\right),H_{p2}\right)$$ (24)

$$t=\text{max}\left(m,\left(T_{x}*Q_{ave}-T_{x}*Q_{1}\right)/Q_{2}\right)$$ (25)

Where,

$P_{1}^{*}$----starting pressure of Generator G1

$P_{2}^{*}$----Starting pressure of generator G2

*k*_1_----Starting pressure coefficient of generator G1

*k*_2_----Starting pressure coefficient of generator G2

*t*----Indicates generator G2 operation time in TX cycle

*t* The calculation principle of $P_{1}^{*}$ is as follows. When *Q*_*ave*_ is large, generator G1 starts earlier, reducing the generator G2 start when the input energy of accumulator is locally large, which is conducive to extending the continuous operation time of generator G1. Avoid frequent startup when *Q*_*ave*_ is started in a small time and late time. The range limit value (*L*_*p*1_, *H*_*p*1_) is given according to the actual situation on site.

The calculation principle of $P_{2}^{*}$ is as follows. When *Q*_*ave*_ is large, generator G2 starts earlier, reducing the overflow valve action when the input energy of accumulator is locally large. When *Q*_*ave*_ is small, it starts later to avoid frequent startup. The range limit value (*L*_*p*2_, *H*_*p*2_) is given according to the actual situation on site.

The calculation principle of *t* is as follows. In order to prevent the operation time from being too short, it indicates the duration of operation of generator G2 in the cycle *T*_*x*_, and the lower limit value is m (s).

After the above parameter calculation is verified to be correct, the control valve set is switched to the electronically controlled working mode, and the power generation system will automatically select the following two working conditions:

In the first working condition, when *Q*_*ave*_ < *Q*_1_, there must be a time period when the generating power is 0. When the oil pressure $P\geq P_{1}^{*}$, the generator G1 operates. According to $P_{1}^{*}$ the calculation principle, the starting time of generator G1 is calculated *Q*_*ave*_ in real time, and the starting time is more reasonable. Making full use of the energy stored in the accumulator can avoid unnecessary starting of G2.

In the second working condition, when the oil pressure *P* is equal to or greater than $P_{1}^{*}$, the generator G1 starts, and when the oil pressure *P* is equal to or greater $P_{2}^{*}$, the generator G2 starts.


$$\text{sgn}(Q_{ave})=\left\{\begin{array}{c} 1,Q_{ave}<Q_{1}\&P\geq P_{1}^{*}\\ 0,Q_{ave}\geq Q_{1}\&P\geq P_{1}^{*} \end{array}\right.$$ (26)

Generator G2 stop conditions are as follows.

1. Once the operation time is *t* and the oil pressure $P<P_{2}^{*}-\Delta P_{2}^{*}$, $\Delta P_{2}^{*}\approx \Delta P_{2}/3$, Δ*P*_2_ is the return error under autonomous working mode, and can be corrected by 1/3 according to the site conditions.
2. Oil pressure $P<P_{2}^{*}-2\Delta P_{2}^{*}$.

Under either of the previously mentioned conditions, generator G2 will cease operation.

## 4 Experimental studies

### 4.1 Simulation of waveform for waves

**Fig 5:**
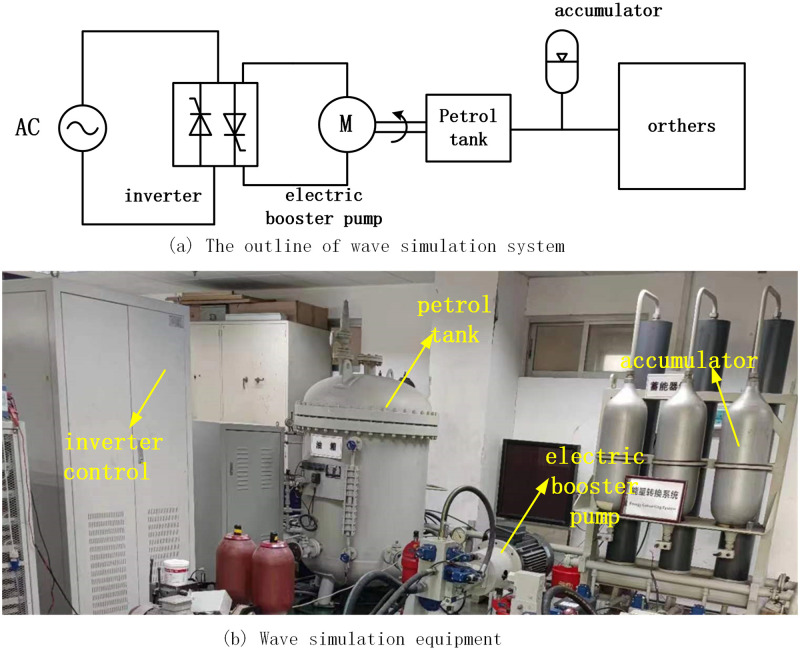
Wave simulation system. (a) The outline of wave simulation system and (b) Wave simulation equipment.

The existing wave-making system covers a large area, has high cost and poor economy. In order to save costs and space, we use a frequency converter and an electric booster pump to create wave forces acting on the wave absorbing floating body and the action of a hydraulic cylinder. The principle is shown in Fig 5. The frequency converter controlled electric booster pump. Then the booster pump pumps the hydraulic oil from the oil tank into the accumulator.

**Fig 6:**
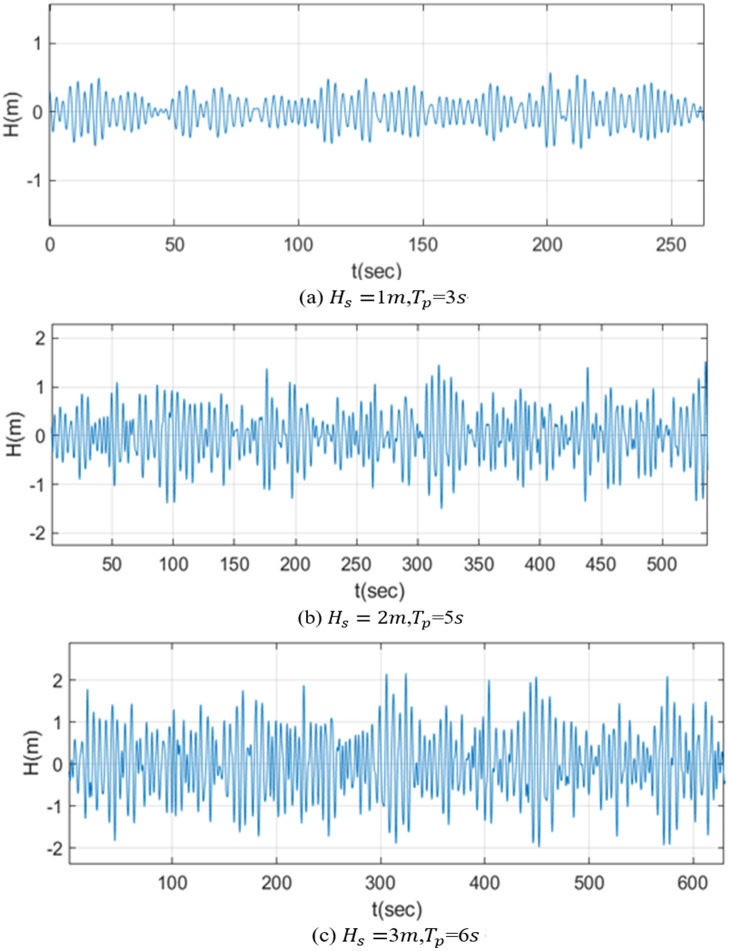
Wave diagram under different conditions. (a) *H*_*s*_ = 1*m*, *T*_*p*_ = 3*s*, (b) *H*_*s*_ = 2*m*, *T*_*p*_ = 5*s*, and (c) *H*_*s*_ = 3*m*, *T*_*p*_ = 6*s*.

The wave generated by the JONSWAP wave spectrum is a composite of an infinite number of linear regular waves, each having distinct amplitudes, periods, wavelengths, and phases. This approach allows for the precise control of the desired wave height and spectral peak period, resulting in a more accurate simulation of the desired waves. Fig 6 displays the waves generated under various significant wave heights and spectral peak periods.

### 4.2 Hydraulic system structure

**Fig 7:**
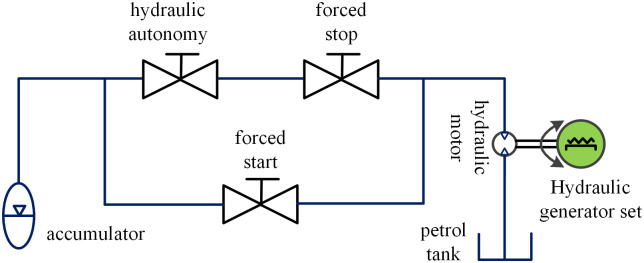
Hydraulic control unit.

The hydraulic energy storage module is comprised of an accumulator, a hydraulic control unit, and a hydraulic motor. The accumulator plays a crucial role in providing a steady output of hydraulic energy, ensuring the stability of the energy output. The hydraulic control unit, as depicted in Fig 7, facilitates various functions such as system debugging, safety protection, system startup, and shutdown.

**Fig 8:**
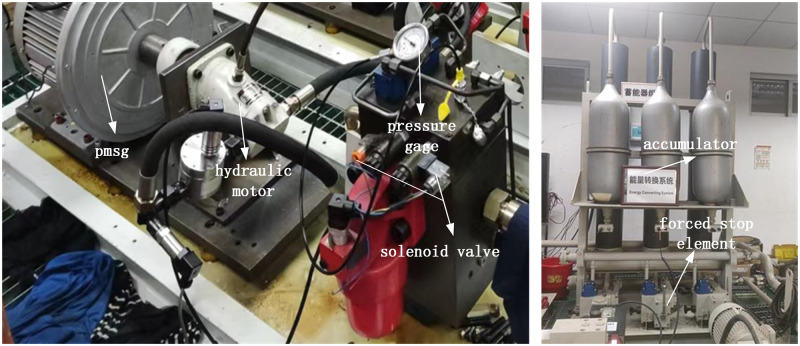
Hydraulic energy storage module.

The hydraulic control unit operates in three distinct modes, hydraulic autonomy, forced stop, and forced work. In hydraulic autonomy mode, the solenoid valve is opened when the experimental system reaches the hydraulic motor’s opening pressure, allowing the hydraulic motor to drive the coaxial generator and generate power under normal working conditions. Forced stop mode is activated in the event of a safety failure, serving as an emergency stop valve for the experimental system. Lastly, forced work mode enables equipment debugging to identify potential sealing issues and verify the normal functioning of the equipment. Fig 8 shows the hydraulic energy storage module.

The solenoid valves of the hydraulic motor can be either opened or closed, and only one switch can be operated (either open or closed) at a time. The forced stop element serves the purpose of redirecting the liquid from the accumulator directly back to the oil tank instead of allowing it to flow into the accumulator. This is particularly useful during maintenance or when the accumulator reaches full pressure.

The electric energy conversion module consists of a permanent magnet synchronous generator and a fully controlled rectifier. The permanent magnet synchronous generator is driven by a coaxial hydraulic motor. The electricity generated by the generator is then rectified by the rectifier and transmitted to the direct-current bus.

### 4.3 Continuous power generation of one storage and two units

**Fig 9:**
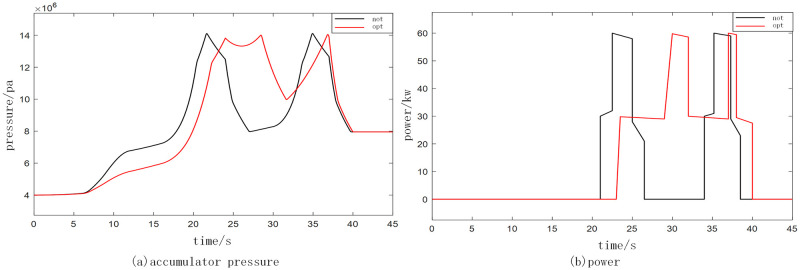
Comparison of small waves before and after optimization. (a) accumulator pressure and (b) power.

**Fig 10:**
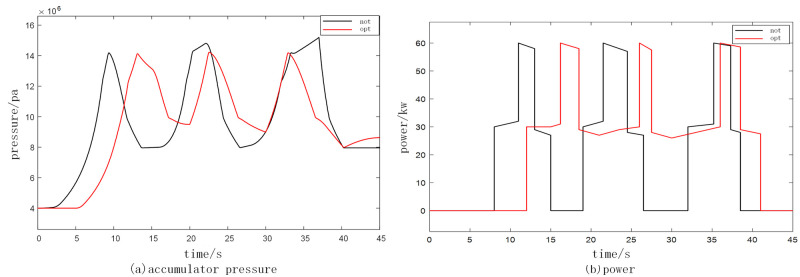
Comparison of medium wave conditions before and after optimization. (a) accumulator pressure and (b) power.

The energy input is partitioned within a system comprising one storage and two units, with the objective of achieving optimal charging through the implementation of a power operation optimization strategy. The simulation parameters utilized are presented in Table 1, while the simulation outcomes for varying wave conditions are illustrated in Figs 9 and 10.

**Table 1:** Simulation parameters.

Equipment	#1 unit	#2 unit
**Accumulator**	Pre inflation pressure: 4bar Volume: 60L	Pre inflation pressure: 4bar Volume: 60L
**Hydraulic motor**	Opening pressure: 14MPa Closing pressure: 10MPa Maximum working pressure: 18Mpa Displacement: 125ml/r Rated speed: 1500r/min	Opening pressure: 12MPa Closing pressure: 8MPa Maximum working pressure: 18Mpa Displacement: 125ml/r Rated speed: 1500r/min
**Alternator**	Rated power: 30KW d. q-axis inductance: 0.45e-3H Internal resistance: 0.005 Ω Flux linkage: 1.0108 Rated speed: 1500r/min Frequency: 50Hz	Rated power: 50KW d. q-axis inductance: 0.15e-3H Internal resistance: 0.0012 Ω Flux linkage: 1.0108 Rated speed: 1500r/min Frequency: 50Hz

Based on the simulation findings, the comparison between the black and red lines indicates that the former represents the pre-optimization state while the latter represents the post-optimization state for both small and medium waves. The accumulator pressure and power before and after optimization are individually evaluated. In the case of small waves, the time taken for power generation from the start is relatively prolonged, resulting in a relatively weak power generation capacity. Conversely, for medium waves, the time required for power generation from the start is relatively shorter, thereby leading to a relatively favorable power generation capacity. The application of the research method in optimizing the accumulator pressure ensures a smooth and uninterrupted power supply throughout the entire generation cycle, yielding commendable optimization outcomes.

## 5 Conclusion

In order to study wave energy power generation, the detailed mathematical models of wave, hydraulic cylinder, accumulator, hydraulic motor and so on are established. In order to verify the accuracy of the simulation model, the corresponding experimental system is designed. In the test system, the functions of the wave absorbing floating body and the hydraulic cylinder are completed by the frequency converter and the electric booster pump. The frequency of the frequency converter is adjusted to obtain the energy input of the required wave conditions. The hydraulic energy storage module has three working modes: Hydraulic autonomy, forced stop and forced work. A new structure of two units driven by a single accumulator is proposed, and the power operation control strategy is designed to solve the problem of power interruption in the single unit wave energy power generation system. By measuring the generating capacity function of the accumulator, the average generating power of the system is calculated in real-time when the control valve group implements the autonomous working mode. Based on this calculated average generating power, the dynamic pressure setting and operating time parameters for the electronic control working mode are determined. Subsequently, the control valve group is switched to the electronic control working mode to precisely regulate the opening and closing of the corresponding hydraulic motor. This enables stable control of generator power generation within the system.
